# Employers’ Response to Workers With Progressive Cognitive Impairment: A Systematic Literature Review

**DOI:** 10.1093/geroni/igab046.2664

**Published:** 2021-12-17

**Authors:** Josephine McMurray, AnneMarie Levy, Logan Reis, Kristina Kokorelias, Jennifer Boger, Arlene Astell

**Affiliations:** 1 Wilfrid Laurier University, Waterloo, Ontario, Canada; 2 Waterloo University, Waterloo, Ontario, Canada; 3 Sunnybrook Research Institute, Toronto, Ontario, Canada; 4 University of Toronto, Toronto, Ontario, Canada

## Abstract

An aging workforce increases the risk of workers experiencing cognitive decline that may lead to a diagnosis of mild cognitive impairment or early onset dementia (MCI|EOD) while still employed. This systematic review explores the use of technologies (defined as any methods, processes, software, hardware or equipment) deployed by employers to accommodate, or build sustainable workspaces for, workers diagnosed with MCI|EOD. After screening 3,860 titles/abstracts and 67 full text reviews, we identified and analyzed eight articles that met our inclusion criteria. We found that: 1) The existing literature almost exclusively focuses on employees’ perspectives on the quality of work life when diagnosed with MCI|EOD, 2) Negative workspace culture toward employees’ cognitive decline, and the variability of disease onset and progression, may account for low employer awareness, 3) Employer responses focus on mitigation of risk associated with workers’ impairment. While this review demonstrates there is scant research exploring employers’ perspectives on employees diagnosed with MCI|EOD, there is even less that explores technologies designed to specifically address employers’ needs and challenges. Technology will increasingly facilitate early identification of progressive neuro-cognitive disorders, and tools to help employers respond to an employee’s MCI|EOD disclosure as a disability accommodation rather than a terminal performance management challenge. Empathic research, that engages organizations in the process of understanding the value of affordable, employer-side technologies that help build diverse, sustainable, productive workspaces is critical to a foundational understanding of our aging workforce and accommodating workers who develop MCI|EOD while still employed.

